# To interact or not to interact: The pros and cons of including interactions in linear regression models

**DOI:** 10.3758/s13428-025-02613-6

**Published:** 2025-02-07

**Authors:** Aljoscha Rimpler, Henk A.L. Kiers, Don van Ravenzwaaij

**Affiliations:** Department of Psychometrics and Statistics, University of Groningen, Grote Kruisstraat 2/1, Heymans Building, room 212, 9712 TS Groningen, The Netherlands

**Keywords:** Moderation, Statistical interactions, Generalizability, Theory

## Abstract

Interaction effects are very common in the psychological literature. However, interaction effects are typically very small and often fail to replicate. In this study, we conducted a simulation comparing the generalizability and estimability of two linear regression models: one correctly specified to account for interaction effects and one misspecified including simple effects only. We manipulated noise levels, predictor variable correlations, and different sets of regression weights, resulting in 9216 different conditions. From each dataset, we drew 1000 samples of *N* = 25, 50, 100, 250, 500, and 1000, resulting in a total of 55,296,000 analyses for each model. Our results show that misspecification can drastically bias regression estimates, sometimes leading to zero or reversed simple effects. Furthermore, we found that when models are generalized to the entire population, the difference between the explained variance in the sample and in the population is often smaller for the misspecified model than for the correctly specified model. However, the comparison between models shows that the correctly specified model explains the data at the population level better overall. These results emphasize the importance of theory in modeling choices and show that it is important to provide a rationale for why interactions are included or excluded in an analysis.

**Supplementary Information:**

The online version contains supplementary material available at 10.3758/s13428-025-02613-6.

Psychological science tries to understand human behavior. To investigate human behavior, researchers often rely on statistical modeling (Borsboom, 2013). Recently, scholars have been arguing for allowing complexity in modeling approaches (Fox et al., 2019; Fried & Robinaugh, 2020; Mathieu et al., 2019; Dubova, 2022) while others have been arguing to keep models simple (Murphy & Russell, 2017; Myung, 2000). The reason for these different perspectives is not purely methodological but also depends on the purpose of the model (Yarkoni & Westfall, 2017). Does one try to explain phenomena by testing or building a theory or does one try to predict phenomena based on the data at hand (Shmueli, 2010)?

Psychological research is often concerned with models that are supposed to explain existing phenomena and the mechanisms behind it (de Rooij & Weeda, 2020; Yarkoni & Westfall, 2017). Popular investigated mechanisms in psychology are *interactions* and *moderations* (Hayes et al., 2017; Murphy, 2021). In the current study, we focus on models that include interactions between quantitative predictors. Interaction models are defined as models in which, in addition to incorporating the predictors in the ordinary additive way, the product term of two predictor variables is incorporated as well (Venables, 1998). By incorporating the interaction term, one can model a dependence of the effect of one predictor on the level of another predictor.

Moderation effects are a special case of interactions in which one variable is of primary interest and researchers are less concerned about the effect of the second variable on its own. However, from a statistical perspective, moderation effects are simply interactions. For a visualization of a moderation effect, see Fig.  1. However, it is unclear whether requirements to detect moderation effects, such as sufficient statistical power, are always met (Aguinis et al., 2005; Murphy & Russell, 2017; Sommet et al., 2022). This led Murphy and Russell (2017) to the conclusion that researchers ought to be very careful when investigating moderation effects or even not investigate them at all.

**Fig. 1:**
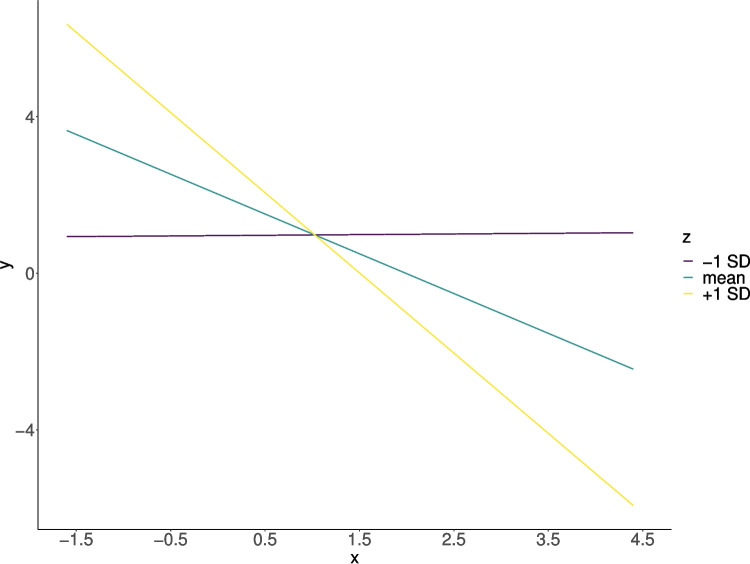
A typical way to visualize moderation effects is through simple slopes. These slopes represent the effect of *X* at different levels of the moderator variable *Z*, showing how the relationship between *X* and *Y* changes depending on the level of *Z*

As pointed out by Yarkoni and Westfall (2017), a robust psychological theory should both explain and predict human behavior. A model that explains behavior accurately captures the psychological mechanism underlying the data. On the other hand, a model that predicts behavior accurately forecasts behavior. Explanatory approaches aim to attain accurate regression weight estimates by minimizing the systematic bias in these estimates, even if this results in a poorer generalizability. In contrast, predictive approaches prioritize reducing the prediction error in unseen data, even if it leads to systematically biased estimates (de Rooij & Weeda, 2020; Yarkoni & Westfall, 2017).

However, more complex models can also generalize better than simpler models when sufficiently large sample sizes are available (Hofman et al., 2017). As the number of candidate variables for a model increases, different selections of variables can perform equally well but lead to substantively different interpretations (Breiman, 2001). This complicates determining which model best reflects reality, particularly in an explanatory context.

These properties should ideally complement each other, but in practice a trade-off between them can arise. It is possible that simpler models, despite not perfectly representing underlying mechanisms, outperform more complex models in predicting outcomes (Wu et al., 2008). This phenomenon occurs because complex models may be overly flexible, risking overfitting to a specific dataset and capturing noise. Simpler models, while potentially having systematically biased estimates due to their theoretical simplifications, may generalize better to new datasets by avoiding overfitting.

However, what causes a model to overfit a dataset? Three different elements are often discussed as affecting a model’s propensity for such over- or underestimations of model fit. These elements are the magnitude of the noise (i.e., variance in the data not explained by the model’s predictions), effect sizes, and (multi-)collinearity. Firstly, the more noise (unexplainable variance) there is, the larger the probability that mainly noise is captured by a model, which results in overfitting. Additionally, the probability of an alternative model capturing the data better is larger.

Secondly, due to the signal-to-noise ratio, smaller true effects or effect sizes increase the risk of overfitting for a given dataset. It is critical to recognize the interdependence of these two factors, as larger noise (i.e., the unexplainable variance) inherently reduces the explainability of a phenomenon by a model and its individual effects, thus reducing effect sizes.

Finally, the presence of (multi-)collinearity, or the correlation structure between predictor variables, could affect the probability that a model overfits a given dataset, due to the overlap of explanation by the individual predictor variables. The combination of these different elements should not only result in overfit but also show in reduced statistical power.

As noted earlier, studies exploring moderation effects are often underpowered (Murphy & Russell, 2017; Sommet et al., 2022) and fail to adequately address several key issues. First, a model including an interaction introduces a kind of non-linearity that is often overlooked. Second, the shape of the interaction is oftentimes not considered, despite its relevance. Third, a persistent misconception exists regarding collinearity, particularly in the context of interaction models. In the following section, we elaborate on this.

## Issue 1: Non-linearity of a moderation model

When one tries to model a moderation effect, one typically uses a linear regression model. The name of this analysis suggests linear relationships between variables. However, introducing an interaction effect (through the product-term $X \times Z$) into the model adds a curvy-linear relationship that is not visible when looking at simple slopes (Finsaas & Goldstein, 2021) in a two-dimensional fashion (see Fig. 1). These relationships are linear in isolation, but when one simultaneously plots the relationship between the predictors X and Z and the outcome Y in a three-dimensional plot, it becomes obvious that the resulting regression plane is not flat, but curved. Non-linearity is not necessarily an issue, however, it becomes problematic if one does not recognize it as such. Additionally, non-linearity can lead to spurious interaction effects (Belzak & Bauer, 2019; Busemeyer & Jones, 1983; Ganzach, 1998; Lubinski & Humphreys, 1990; Rimpler et al., 2024).

## Issue 2: Shape of interaction

It has been acknowledged that the shape of the interaction is crucial when it comes to the power to find a significant interaction (Finsaas & Goldstein, 2021; Baranger et al., 2023; Afshartous & Preston, 2011). The shape of an interaction is defined by the effect each individual predictor (including the moderation term) has on the outcome variable, thus by the signs and magnitude of each individual predictor. Two different approaches are used to describe the shape of interaction effects.

The first approach looks at the ratio between one main effect and the moderation effect and investigates the cross-over points, thus focusing on the simple slopes relative to each other (see, e.g., Baranger et al. (2023)). In this context, people typically talk about attenuated interactions (i.e., smaller interaction effect than simple effects, all simple slopes pointing into the same direction), knock-out interactions (i.e., interaction effect has the same regression coefficient as one of the simple effects, slope of one simple effect becomes 0 at mean), and cross-over interactions (i.e., interaction effect is larger than simple effect, effects go into different directions), see Fig. 1 for a visualization of simple slopes. For a more detailed review of this perspective, see e.g., Baranger et al. (2023); McCabe and King (2023); Giner-Sorolla (2018). A disadvantage of this typology is that one needs to clearly define the *moderating* variable, while the assignment of which variable is the moderator is arbitrary.

**Fig. 2:**
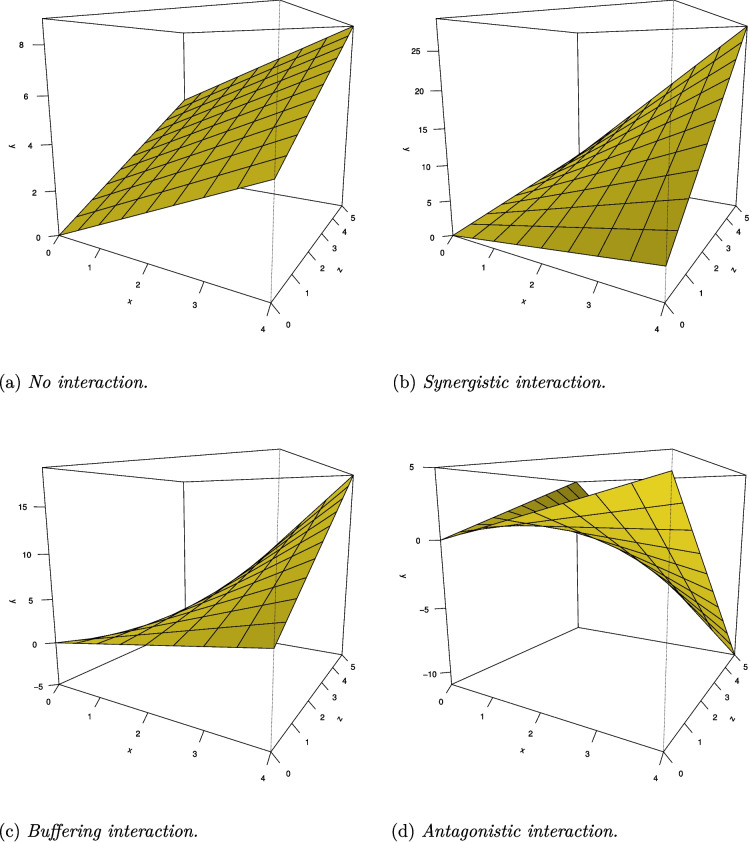
The nonlinear relationship between predictor and outcome variables when an interaction term is included. The relationship between *X* and *Y* is linear and the relationship between *Z* and *Y* is linear. However, there is non-linearity when looking at the combination of *X* and *Z* jointly and *Y*. Different combinations of regression weights can lead to different interaction types. **a** No interaction; **b** Synergistic interaction: both predictors and their interaction (i.e., *X*, *Z*, and $X \times Z$) all have the same sign (here: $\beta _X$ = 1, $\beta _Z$ = 1, $\beta _{XZ}$ = 1); **c** Buffering interaction: simple predictors have opposing signs (here: $\beta _X$ = 1, $\beta _Z$ = -1, $\beta _{XZ}$ = 1); **d** Antagonistic interaction: simple predictors and the interaction have opposite signs (here: $\beta _X$ = 1, $\beta _Z$ = 1, $\beta _{XZ}$ = -1)

The second approach takes the perspective of the whole model. In this perspective, the moderator can enhance the effects of the simple effects through a synergistic interaction (i.e., all signs go in the same direction), can have a mixed effect of one predictor through a buffering interaction (i.e., simple effects have differing signs), or can reduce the effects of the simple effects through an antagonistic interaction (i.e., simple effects go in the direction opposite to that of the moderation effect), for a more detailed review, see (McCabe & King, 2023). Important to note is that a theoretical interaction ranging from minus infinity to infinity will always be shaped like an antagonistic interaction, with one saddle point and counteracting effects. For a visualization of different interaction types when all variables take on only positive values, see Fig. 2. Throughout this article, we will use the terminology of the second approach.

A hypothetical synergistic interaction is the interaction between the independent variables *hours of sleep* and *hours of studying*, on the dependent variable *exam result*. Both *hours of sleep* and *hours of studying* have a positive effect on *exam result*, but the combination of both has an additional positive (synergistic) effect on *exam result*. A hypothetical antagonistic interaction is the interaction between the independent variables *sugar content* and *fat content*, on the dependent variable *ice cream taste*. Both *sugar content* and *fat content* have a positive effect on *ice cream taste*, however, if *sugar content* and *fat content* are both high the *ice cream taste* becomes worse (cf. Rouder et al. (2016)). A hypothetical buffering interaction is the interaction between the independent variables *salt* and *pasta*, on the dependent variable *taste*. A dish containing just salt will taste bad (it is just salty), a dish containing just pasta will taste okay (if a bit bland). Combining *salt* with *pasta* has a positive influence on the *taste*, despite the fact that salt by itself has a negative effect.

The shape of the interaction adds a layer of complexity to the data that is often not acknowledged, and is also not captured in typical effect size measures such as $f^2$ (Cohen, 1988, p. 426). This makes it more likely that study planning goes wrong (e.g., in determining the statistical power). The shape of the interaction also influences the extent of model misspecification. If a synergistic interaction is present, and one incorrectly applies a linear regression with only *X* and *Z*, and not the interaction, the model still performs reasonably well because all components of the data point roughly in the same direction. However, if an antagonistic interaction is present, the misspecification by a model with only *X* and *Z* can lead to a situation where the underlying interaction is of the same size as one of the underlying simple effects (*X* or *Z*) and roughly cancels it out, and as a consequence, when using the misspecified model one will only (or mainly) find an effect of the other predictor.

## Issue 3: Misconception of collinearity in the case of moderation

The influence of collinearity, the correlation between the predictor variables, on moderation models has been discussed by Dalal and Zickar (2012); Disatnik and Sivan (2016); Iacobucci et al. (2016); Olvera Astivia and Kroc (2019); Afshartous and Preston (2011). Nonetheless, we think it is important to shortly elaborate on collinearity in the case of moderators because there seem to be common misunderstandings. Generally, collinearity between predictors inflates the standard errors of the regression coefficients and leads to numerically unstable coefficients. However, maybe counterintuitively, this is not the case for the collinearity introduced by an interaction term (Iacobucci et al., 2016; Olvera Astivia and Kroc, 2019; Afshartous & Preston, 2011; Aguinis et al., 2017; Disatnik & Sivan, 2016). Indeed, introducing a moderator as a predictor inherently introduces a correlation between predictor variables, since the product term $X \times Z$ is calculated based on *X* and *Z* and therefore is correlated with both of the predictors. As a way of dealing with collinearity in moderation analyses, mean centering of the predictors before taking their product has been proposed (Cohen et al., 2015, pp. 261). Mean centering is the procedure where one subtracts the mean of a variable from the variable itself, thus centering the variable around its mean. However, it turns out that no such measure is needed to deal with this because even though there is a correlation between the predictor variables and the interaction term, this correlation does not affect the stability of the estimates of the interaction term (Afshartous & Preston, 2011; Aguinis et al., 2017; Disatnik & Sivan, 2016). Hence, as has been discussed by Afshartous and Preston (2011), the collinearity introduced by an interaction effect does not influence the power of finding an interaction effect. Olvera Astivia and Kroc (2019); Echambadi and Hess (2007), discussed the effects of mean centering and showed that this procedure does not change the estimated interaction effect and its precision, and neither the $R^2$ for the whole model. Importantly, the standard errors of the simple effects need to be interpreted after mean centering because they now reflect the precision of the regression coefficients for the mean-centered, and hence altered, predictors.

Summarizing, mean centering can lead to a more interpretable model, however it will not affect the magnitude or stability of the found interaction effect. The standard error for the simple effects have a changed interpretation after mean centering and thus smaller standard errors do not necessarily reflect a more stable estimate of the simple effects for the predictors in their original interpretation.

## Current study

We used a simulation to examine the influence of the previously mentioned factors when investigating interaction effects. In the current study we conducted a simulation to examine the generalizability of linear regression models, when differently sized interaction effects in the data exist. Two different linear regression models are applied – the correct model, which covers the interaction effect, and the misspecified model with only simple effects, hence not accounting for the interaction term underlying the data. The current work is guided by three research questions:

1. How is the power to find significant regression weights for the predictors and their interaction influenced by noise, collinearity, and the shape of the interaction for the correctly specified model as well as a misspecified model that does not account for the interaction?
2. Under what circumstances does the misspecified model perform (appreciably) worse in terms of out-of-sample prediction than the correct model?
3. Are there situations in which parameter estimates of the misspecified model lead to better predictions in the population than predictions based on parameter estimates of the correctly specified model?

## Method

### Data generation

Data generation and analyses were performed in the R programming environment (R Core Team, 2021). All materials to reproduce the study can be found on https://osf.io/myq8u/. The simulations that we conducted are described below.

In a first step, we created large finite population data sets, each with $N = 10^6$, for each set of varied parameters. This was accomplished by creating variables *X* and *Z* that follow a normal distribution with means equal to 1 and 2, respectively, and $SD = 1$. The two means were chosen to be non-zero, different from each other, and of a similar magnitude, to reflect a realistic situation. The outcome variable *Y* was created according to the following equation:

1 $$\begin{aligned} {\begin{matrix} Y^* & =\beta _0+\beta _1X+\beta _2Z+\beta _3XZ \\ Y & =\beta _0+\beta _1X+\beta _2Z+\beta _3XZ+\varepsilon \end{matrix}} \end{aligned}$$

where $\varepsilon \sim \mathcal {N}(0,\,\lambda \sigma (Y^*))$ and $\lambda$ is the noise level.

We set the intercept $\beta _0$ to zero for all conditions. The weights $\beta _1$ and $\beta _2$ varied from -1 to 1 in steps of 0.25 excluding 0, while $\beta _3$ was varied likewise and did include 0, representing no interaction effect on the population level. This variation indirectly varied the effect size $f^2$ of the interaction effect and its shape. The $f^2$ is a standardized effect size measure for an interaction effect and is computed as the $R^2$ difference between two models (i.e., a model 2 including and model 1 excluding an interaction effect; Cohen, 1988, p. 411), thus

2 $$\begin{aligned} {\begin{matrix} f^2 & =\frac{R^2_{model2}-R^2_{model1}}{1-R^2_{model2}}\\ \end{matrix}} \end{aligned}$$

Additionally, we varied the noise level $\lambda$, which indirectly influences the effect size $f^2$ of the interaction effect. The noise level was adjusted to yield $R^2$ values of .1, .3, .5, and .7, respectively, determined by the equation

3 $$\begin{aligned} \lambda =\sqrt{\frac{1}{R^2}-1} \end{aligned}$$

Furthermore, we varied the correlation between the two predictor variables *X* and *Z* to investigate whether collinearity influences the generalizability of interaction effects. All resulting parameter values are shown in Table  1.

**Table 1:** Parameter space

Parameter				
Noise level $\lambda$	$r(X,Z)$	$\beta _1$	$\beta _2$	$\beta _3$
3.0	0.75	-1.0	-1.0	-1.0
1.53	0.5	-0.75	-0.75	-0.75
1.0	0.25	-0.5	-0.5	-0.5
0.65	0.0001	-0.25	-0.25	-0.25
		0.25	0.25	0.00
		0.5	0.5	0.25
		0.75	0.75	0.5
		1.0	1.0	0.75
				1.0

Parameter values were chosen in such a way that the resulting effect sizes for the interactions cover the very small effect sizes Aguinis et al. (2005) have reported to be found in practice (median $f^2$ = 0.009), as well as the commonly considered small ($f^2$ = .02), medium ($f^2$ = 0.15) and large effect ($f^2$ = 0.35) sizes (Cohen, 1988, p. 426). To confirm a priori that the chosen parameter values cover the $f^2$ range of interest, we have run an a priori simulation with a different seed on the same parameter space in which we only estimated $f^2$ of the interaction effect on the full populations. While there are populations that result in unreasonably large $f^2$s ($f^2$ > 0.5) this was only the case for 3% of the populations. The 5th and 95th percentile of the $f^2$ were 0.00 and 0.39.

From each population dataset, a total of 1000 samples per sample size *N* were drawn (*N* = 25, 50, 100, 250, 500, and 1000). On each sample, we fitted two different regression models. One with only the predictors *X* and *Z* (from here on: “simple effects model”), and a second regression model with predictors, *X*, *Z* and the interaction $X \times Z$ (from here on: “interaction model”).

### Measures

To investigate the performance of the models applied, we used various different measures. We assessed the explanatory capacities of each model using the following metrics:

- bias of the (estimated parameters in the) model, measured as the difference between the true regression weight $\beta$ and estimated regression weight *b*. We used this to evaluate how well the model estimates the true underlying parameters.
- power to find significant effects of the different regression components, measured as the proportion of significant findings at an $\alpha$-level of 0.05. We used this to evaluate the model’s ability to detect effects of different regression components when present.

We assessed the predictive capacities of each model using the following metrics:

- generalizability, measured as the explained variance when the estimated model for the sample is applied to the full population. We used this to assess the model’s performance in predicting outcomes for the entire population based on the model fitted to the sample.
- overfit, measured as the difference between $R^2$ in the sample and $R^2$ when the sample estimated model gets generalized to the full population. We used this to assess to what extent the sample $R^2$ may be too optimistic of an estimate of the explained variance in the population.
- difference in generalizability, measured as the difference between $R^2$ when sample estimates are generalized to the full population for the two different models. This measure serves to identify the relative magnitude of over- and underfitting when the models are applied to the full population.

## Results

### Bias of regression estimates

One of the key principles in ordinary least-squares regression is that, under correct representation of the data, the regression estimates are unbiased given a sufficiently large sample size. Therefore, understanding the bias (*B*) of regression estimates becomes particularly important when the model is misspecified. When the data-generating mechanism is known, we can calculate the bias of the estimates for the misspecified model considering only intercept, X and Z, and not modeling the interaction. This is expressed by the following equations (see Supplement for the derivation):

4 $$\begin{aligned} b_{e0} =\beta _0-\beta _3\mu _z\mu _x+\beta _3r_{xz} \end{aligned}$$

5 $$\begin{aligned} b_{e1} =\beta _1+\beta _3\mu _z \end{aligned}$$

6 $$\begin{aligned} b_{e2} =\beta _2+\beta _3\mu _x \end{aligned}$$

7 $$\begin{aligned} B_{n} =b_{en}-\beta _n \end{aligned}$$

Here, subscript *e* denotes the regression weight we would get if we would analyze the population data; *n* denotes the predictor variable; and $\mu$ denotes the population mean of the respective variable. As we show in our derivation, this also gives the statistically expected regression weights. An illustration of expected regression weights for a misspecified model is provided in Table 2. In this example, we set parameters as follows: $\beta _0=0$, $\beta _1=1$, $\beta _2=1$, $r_{xz}=0.0001$, $\mu _x=1$, $\mu _z=2$. We varied the parameter $\beta _3$ to demonstrate its influence on the regression estimates.

One can see that the expected regression estimates vary systematically as a function of $\beta _3$. For positive values of $\beta _3$, the expected estimates $b_1$ and $b_2$ have the same sign as $\beta _3$, whereas the estimated $b_0$ has the opposite sign. Note that the true value of $\beta _1$ and $\beta _2$ is 1; we see that depending on the true value of $\beta _3$ the expected estimates of $b_1$ and $b_2$ can be 0 or even negative for increasingly lower values of $\beta _3$.

The cases where the expected estimates of $b_1$ or $b_2$ are 0 (highlighted in italics) are in particular interesting in a Null-Hypotheses testing framework. In these cases, we expect that one will rarely find significance for these simple effects. Intuitively, one might even think, since here the expectation of the regression weight is 0, significance would be found in only $\alpha *100$% of the times. While this does not hold true (see section “Caution when interpreting *p* values in misspecified models”), it does happen relatively rarely in such cases as can be seen in our simulation. Thus, in these cases, a researcher is likely to conclude that there is no simple effect of the relevant independent variable on the dependent variable. The cases where the expected estimates of $b_1$ or $b_2$ have a sign opposite to the true population parameters are even more concerning, as a researcher might confuse a positive effect on the dependent variable for a negative one, or vice versa.

### Power

In the previous section, we described how fitting a model without an interaction to a data set for which an interaction exists can lead to regression estimates of one of the simple effects being (close to) 0, when the true parameters are not 0. In this section, we want to showcase how this affects the power to find these regression estimates for some of the simulated conditions.

**Fig. 3:**
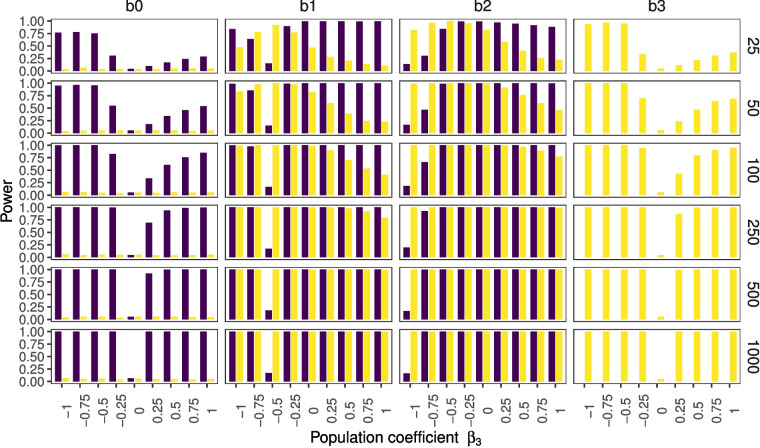
*p* values for non-centered data, where the $noise level = 0.65$ and $r_{x,z}=0.0001$. The figure depicts the percentages of significant findings for the different regression estimates on the *y*-axis across different population parameters $\beta _3$, indicating the interaction, on the *x*-axis. *Purple bars* correspond to the simple effects model; *yellow bars* correspond to the interaction effects model

Figure 3 shows results for population parameters that are the same as in the previous section (see Table 2). This figure shows how the misspecification of the simple effects model compensates for the lack of an interaction effect. When $\beta _3 = -1$ we find 13-19% significant regression estimates $b_2$ (for different sample sizes) and when $\beta _3 = 0.5$ we find 15-19% significant regression estimates $b_1$. Note that these figures deviate from the nominal type 1 error rate of 0.05 (more on this in the next section). Furthermore, the tipping point for the $b_1$ estimates is visible because when $\beta _3 = -0.5$ the power increases for smaller and larger values of $\beta _3$ (but recall that the estimate of the effect will have the wrong sign in case $\beta _3$ gets smaller).

**Fig. 4:**
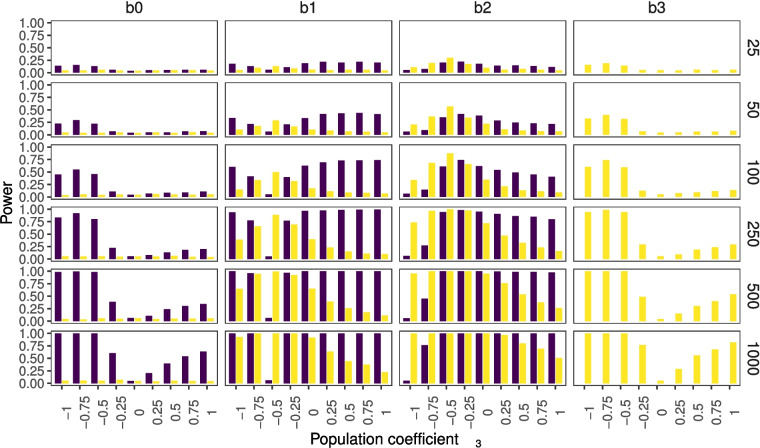
*p* values for non-centered data, where the $noise level = 3$ and $r_{x,z}=0.0001$. The figure depicts the percentages of significant findings for the different regression estimates on the *y*-axis across different population parameters $\beta _3$, indicating the interaction, on the *x*-axis. *Purple bars* correspond to the simple effects model; *yellow bars* correspond to the interaction effects model

**Fig. 5:**
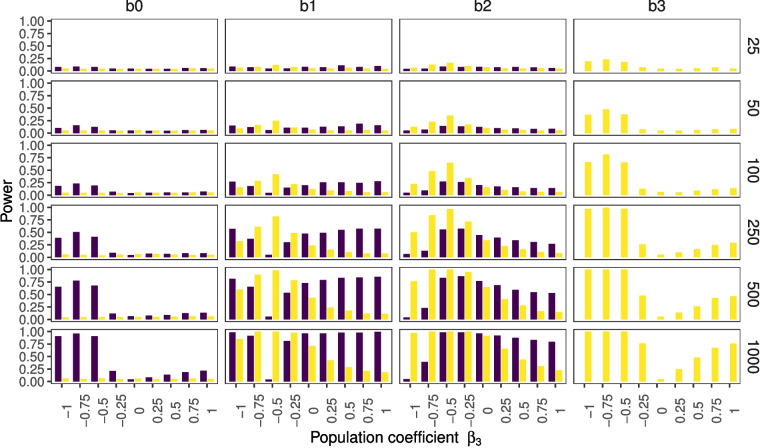
*p* values for non-centered data, where the $noise level = 3$ and $r_{x,z}=0.75$. The figure depicts the percentages of significant findings for the different regression estimates on the *y*-axis across different population parameters $\beta _3$, indicating the interaction, on the *x*-axis. *Purple bars* correspond to the simple effects model; *yellow bars* correspond to the interaction effects model

Figure 4 provides an overview of the results with a noise parameter of $\varepsilon =3$. Figure 5 shows the results for the conditions in which we additionally set the correlation between *X* and *Z* to $r = 0.75$. The comparison of these three different simulation conditions shows that statistical power is strongly influenced by sample size (compare rows within figures), and moderately influenced by noise (compare Figs.  3 and  4). The power to find an interaction effect is only weakly influenced by collinearity (compare Figs.  4 and  5). The comparison of the latter figures also shows that the power to find the simple effects decreases with an increase in collinearity. Furthermore, it is visible that in the special cases of $\beta _3 = -1$ and $\beta _3 = -0.5$ the rate of significant estimates for $b_1$ and $b_2$ approaches 5%.

These results allow for an examination of the effect of the type of interaction on statistical power. Recall that the combination of a positive $\beta _1, \beta _2$ and $\beta _3$ parameter represents a synergistic interaction, and the combination of a positive $\beta _1$ and $\beta _2$ parameter with a negative $\beta _3$ parameter results in an antagonistic interaction. Furthermore, the special cases are visible where the bias due to misspecification cancels out one simple effect when $\beta _3=-1$ or the other simple effect when $\beta _3=-0.5$. Comparing the antagonistic interactions (left side of 0) to the synergistic interactions (right side of 0) it is visible that the power is higher for antagonistic compared to synergistic interactions (i.e., within each panel, the *yellow bars* are higher on the left-hand side than on the right-hand side).

**Table 2:** Expected parameter estimates for misspecified model with only intercept, *X*, and *Z*, when in the population $\beta _0=0$, $\beta _1=1$, $\beta _2=1$, $r_{xz}=0.0001$, $\mu _x=1$, $\mu _z=2$, $\lambda =0.65$, across different $\beta _3$ values

$\beta _3$	$b_{e0}$	$b_{e1}$	$b_{e2}$
–1	2.0	–1.0	*0.0*
–0.75	1.5	–0.5	0.25
–0.5	1.0	*0.0*	0.5
–0.25	0.5	0.5	0.75
**0.0**	**0.0**	**1.0**	**1.0**
0.25	–0.5	1.5	1.25
0.5	–1.0	2.0	1.50
0.75	–1.5	2.5	1.75
1.0	–2	3.0	2.00

The values in boldface show unbiased estimates, values highlighted in italics show cases where the expected estimate is 0, and thus will not be detected when tested against 0. It is important to note that while the misspecification is always problematic, it is arguably worse in some situations compared to others. For instance, in the column with $b_1$ values, it is visible that the first two entries are negative and the third entry is zero. For these cases, the qualitative as well as the quantitative conclusions about this variable are wrong. The remaining entries show effects that go in the correct direction, but they still differ in magnitude compared to the true effect

### Caution when interpreting *p* values in misspecified models

As visible in Table 2, the regression coefficients $b_1$ and $b_2$ are 0 in the special cases of $\beta _3= -0.5$ and $\beta _3= -1$ respectively, thus we would expect a rate of significant findings according to our $\alpha$-level of 0.05, across noise and collinearity conditions. Figure 3 shows clear deviations from this. However, type 1 error rate is not only affected for these special cases, but for all misspecified cases. In the following we will elaborate on this. The data generation can be divided into two components:

8 $$\begin{aligned} Y=\beta _1X+\beta _2Z+\beta _3XZ+\varepsilon \end{aligned}$$

Part I:

9 $$\begin{aligned} Y(X,Z) = \beta _1X+\beta _2Z \end{aligned}$$

It is visible that Part I can be easily regressed on *X*, *Z* or both, since nothing in Y(X,Z) cannot be explained by *X* or *Z*.

Part II:

10 $$\begin{aligned} Y(XZ)=\beta _3XZ+\varepsilon \end{aligned}$$

Applying a regression of *Y*(*XZ*) on *X* and *Z* will partially explain the second part of *Y*. The linear regression will give a result:

11 $$\begin{aligned} \hat{Y}(XZ)=a+bX+cZ \end{aligned}$$

Thus, the residuals are

12 $$\begin{aligned} Y(XZ)-\hat{Y}(XZ)=\beta _3XZ+\varepsilon -(a+bX+cZ) \end{aligned}$$

This means, unless

13 $$\begin{aligned} a+bX+cZ-\beta _3XZ=0 \end{aligned}$$

the assumption of independent error and also the assumption of homoscedasticity and normality of the residuals are not met in case of a misspecified model. A violation of the assumption of independent error and homoscedasticity has been shown to be influential, even with large sample sizes (Schmidt & Finan, 2018). This effect can be expected to be relatively small if the well-behaved part of the residuals (i.e.,$\varepsilon$) dominates the other not-well-behaved part. This happens with an increase in the noise level $\lambda$, and can indeed be seen to happen in Fig. 5, as we see that the power gets closer to the nominal 5% type I error rate. It also happens with an increase in the correlation between *X* and *Z* (comparison Figs. 3 and 4).

### Comparison of generalizations

A typical measure to assess a linear regression model is the proportion of variation in the outcome variable explained by the predictor variables, which is commonly referred to as $R^2$. In the current work, we use different variants of $R^2$ related measures, which is why we refer to the regular $R^2$ as $R^2(s)$, where the *s* indicates the sample.

First, we calculated the difference between $R^2(s)$, which should always be larger for the interaction effect model and is calculated as $R^2(s)_{interaction}-R^2(s)_{simple}$. This measure does not say anything about the generalization, but it can serve as a baseline for comparisons and is the measure researchers typically obtain when they compare outcomes of two linear regression models, and is often denoted as $R^2(s)_{change}$.

When a model fits the sample data well, but generalizes poorly to the population, it usually indicates model overfit. To quantify the generalization of a model from the sample to the population, we used the regression estimates obtained from a sample, applied it to the full population (i.e., the ‘parent’ sample with $N=10^6$) and calculated the resulting $R^2$, which we denote with $R^2(p)$.

Note that in this simulation study, we have the luxury of being able to use $R^2(p)$. In practice, the calculation of this measure is typically not possible due to the unavailability of the full population. As a result, one typically approximates $R^2(p)$ through cross-validation methods or the adjusted $R^2$. In the appendix, we illustrate an example of a proxy for $R^2(p)$ obtained via the leave-one-out cross-validation procedure.

Obtaining $R^2(p)$ for both models allows the direct comparison of a model’s capability to explain the data at the population level, and thus the generalizability of the model. Here, we refer to the comparison of the $R^2(p)$ between models as $\Delta R^2(p)$:

14 $$\begin{aligned} \Delta R^2(p) =R^2(p)_{interaction}-R^2(p)_{simple} \end{aligned}$$

In addition to comparing the generalizability of both models, we also calculate the degree of overfit as $R^2(s,p)$:

15 $$\begin{aligned} R^2(s,p) =R^2(s)-R^2(p) \end{aligned}$$

We can compare $R^2(s,p)$ between models by calculating $\Delta R^2(s, p)$:

16 $$\begin{aligned} \Delta R^2(s,p) =R^2(s,p)_{interaction }-R^2(s,p)_{simple } \end{aligned}$$

The results of these different comparisons are shown in Tables 3 and 4. Table 3 shows the comparison across noise conditions, whereas Table 4 shows the comparison across sample sizes. Ideally, $R^2(s, p)$ values should be low, or close to zero, so positive $\Delta R^2(s, p)$ values favor the simple effects model, while negative values favor the interaction effect model. Ideally, $R^2(p)$ values should be high, or close to one, so positive $\Delta R^2(p)$ values favor the interaction model, while negative values favor the simple effect model. The *k* in the table refers to the number of analyses that are compared per row.

**Table 3:** Comparison of the difference of $R^2$ in sample and population across type of models and across noise levels

	$\Delta R^2(s)$			$\Delta R^2(s,p)$				$\Delta R^2(p)$			
Noise $\lambda$	$Q_{0.1}$	Mdn	$Q_{0.9}$	$Q_{0.1}$	Mdn	$Q_{0.9}$	%$<0$	$Q_{0.1}$	Mdn	$Q_{0.9}$	%$<0$
Synergistic				k = 3.072.000							
0.65	0.01	**0.04**	0.08	-0.03	**0.00**	0.04	55	0.01	**0.04**	0.07	5
1.0	0.00	**0.03**	0.07	-0.02	**0.00**	0.05	50	0.00	**0.03**	0.05	11
1.53	0.00	**0.02**	0.06	-0.01	**0.00**	0.07	44	-0.02	**0.01**	0.03	19
3.0	0.00	**0.01**	0.04	0.00	**0.00**	0.09	34	-0.05	**0.00**	0.01	36
Antagonistic				k = 3.072.000							
0.65	0.09	**0.25**	0.52	-0.16	**-0.02**	0.05	65	0.12	**0.28**	0.57	0
1.0	0.05	**0.18**	0.38	-0.12	**-0.01**	0.07	60	0.08	**0.20**	0.40	1
1.53	0.03	**0.11**	0.25	-0.07	**-0.01**	0.08	56	0.03	**0.11**	0.24	4
3.0	0.00	**0.04**	0.11	-0.03	**0.00**	0.09	48	-0.01	**0.03**	0.08	14
Buffering				k = 6.144.000							
0.65	0.03	**0.08**	0.17	-0.05	**-0.01**	0.04	59	0.03	**0.09**	0.16	2
1.0	0.01	**0.06**	0.13	-0.04	**0.00**	0.06	54	0.02	**0.06**	0.12	5
1.53	0.01	**0.04**	0.10	-0.02	**0.00**	0.07	49	0.00	**0.03**	0.07	11
3.0	0.00	**0.01**	0.06	-0.01	**0.00**	0.09	40	-0.04	**0.01**	0.02	26
No Interaction				k = 1.536.000							
0.65	0.00	**0.00**	0.01	0.00	**0.00**	0.03	6	-0.02	**0.00**	0.00	89
1.0	0.00	**0.00**	0.02	0.00	**0.00**	0.05	6	-0.03	**0.00**	0.00	89
1.53	0.00	**0.00**	0.03	0.00	**0.00**	0.07	6	-0.04	**0.00**	0.00	89
3.0	0.00	**0.00**	0.03	0.00	**0.00**	0.09	6	-0.05	**0.00**	0.00	89

**Table 4:** Comparison of the difference of $R^2$ in sample and population across types of models, and across sample sizes used

	$\Delta R^2(s)$			$\Delta R^2(s,p)$				$\Delta R^2(p)$			
N	$Q_{0.1}$	Mdn	$Q_{0.9}$	$Q_{0.1}$	Mdn	$Q_{0.9}$	%$<0$	$Q_{0.1}$	Mdn	$Q_{0.9}$	%$<0$
Synergistic				k = 2.048.000							
25	0.00	**0.02**	0.12	-0.03	**0.02**	0.22	35	-0.13	**0.00**	0.06	47
50	0.00	**0.02**	0.09	-0.02	**0.01**	0.09	42	-0.02	**0.01**	0.06	31
100	0.00	**0.02**	0.07	-0.02	**0.00**	0.05	46	-0.01	**0.02**	0.05	18
250	0.00	**0.02**	0.06	-0.01	**0.00**	0.02	50	0.00	**0.02**	0.05	7
500	0.00	**0.02**	0.06	-0.01	**0.00**	0.02	51	0.00	**0.02**	0.05	3
1000	0.00	**0.02**	0.05	-0.01	**0.00**	0.01	51	0.00	**0.02**	0.05	1
Antagonistic				k = 2.048.000							
25	0.01	**0.11**	0.36	-0.21	**-0.02**	0.22	57	-0.06	**0.13**	0.45	18
50	0.01	**0.12**	0.36	-0.14	**-0.02**	0.11	60	0.01	**0.14**	0.41	8
100	0.02	**0.13**	0.36	-0.09	**-0.01**	0.08	59	0.02	**0.14**	0.39	3
250	0.03	**0.13**	0.36	-0.06	**-0.01**	0.05	57	0.03	**0.14**	0.37	0
500	0.03	**0.13**	0.36	-0.04	** 0.00**	0.03	55	0.03	**0.14**	0.37	0
1000	0.03	**0.14**	0.36	-0.03	** 0.00**	0.02	54	0.03	**0.14**	0.37	0
Buffering				k = 4.096.000							
25	0.00	**0.04**	0.17	-0.07	**0.01**	0.22	44	-0.11	**0.03**	0.13	34
50	0.00	**0.04**	0.14	-0.05	**0.00**	0.10	49	-0.02	**0.04**	0.12	19
100	0.00	**0.04**	0.13	-0.04	**0.00**	0.06	52	0.00	**0.04**	0.11	9
250	0.01	**0.04**	0.12	-0.02	**0.00**	0.03	53	0.01	**0.04**	0.11	3
500	0.01	**0.04**	0.11	-0.02	**0.00**	0.02	53	0.01	**0.04**	0.11	1
1000	0.01	**0.04**	0.11	-0.01	**0.00**	0.01	52	0.01	**0.05**	0.11	0
No Interaction				k = 1.024.000							
25	0.00	**0.01**	0.07	0.00	**0.03**	0.22	12	-0.16	**-0.02**	0.00	83
50	0.00	**0.01**	0.03	0.00	**0.01**	0.09	9	-0.05	**-0.01**	0.00	85
100	0.00	**0.00**	0.02	0.00	**0.01**	0.04	7	-0.02	** 0.00**	0.00	88
250	0.00	**0.00**	0.01	0.00	**0.00**	0.01	5	-0.01	** 0.00**	0.00	91
500	0.00	**0.00**	0.00	0.00	**0.00**	0.01	3	0.00	** 0.00**	0.00	93
1000	0.00	**0.00**	0.00	0.00	**0.00**	0.00	3	0.00	** 0.00**	0.00	95

The $\Delta R^2(s)$ values in Tables 3 and 4 show that on sample level the $R^2$ is, as long as an interaction effect exists in the data, always larger for the model including an interaction effect, which needs to be the case since $R^2$ cannot be smaller with added predictors. However, sizable differences are visible between the different shapes of interaction effects. In the case of a synergistic interaction, adding the interaction only yields small differences. Contrary to this, adding an interaction effect in the antagonistic case can lead to substantial changes in $R^2$. The buffering interaction is somewhere in between, with a median $R^2$ difference of 0.04. Table 3 indicates that the degree of overfit as well as effect sizes of the interaction effect depend on the noise level as well as the shape of the interaction.

Furthermore, the results show that with an increase in sample size the differences of overfit become smaller, across interaction shapes, for both models. The clearly positive $\Delta R^2(s,p)$ values, as mainly found for the small sample sizes, refer to cases where there is considerably more overfitting with the interaction model than with the simple effects model. This is not the case for antagonistic interactions, which is expected since antagonistic interaction effects have the strongest bending (see Fig. 2), indicating modeling interaction effects is definitely needed in such cases, and does not leave room for excessive overfitting.

Inspecting $\Delta R^2(p)$, we can verify in which cases the interaction model actually works better in the population than the simple model, or conversely, where the interaction model actually does not, and only fits better than the simple effects model in the sample. The latter cases are particularly interesting because in practice one only has the $R^2(s)$ for the sample and one might mistakenly conclude that the interaction model is best in general, while it only is in the sample, and not in the population. Therefore, we also computed in what percentage of cases this happens. It is visible that only for synergistic interactions with $N = 25$ and no interactions with smaller sample sizes does the simple effects model as often as not yield better results for the generalization than the interaction model. Also, and not surprisingly, if there is no interaction in the model underlying the data, then the simple effects model-based estimates very often work better in the population than the ones obtained with the interaction model. For all other cases, however, even though $R^2(s)$ is often closer to $R^2(p)$ for the simple effects models, $R^2(p)$ tends to favor the interaction effect models. Nevertheless, in quite a few conditions, there is a sizeable percentage of cases in which the main effects model does work better in the population than the interaction effects model. This indicates that, while the difference between the $R^2$ found in the sample is closer to the $R^2$ in the population for the simple effects model, the interaction effect model is oftentimes favored in the population. The difference is smallest in the case of a true synergistic interaction effect, particularly for smaller sample sizes. In those cases, the proportion of better generalizing simple effect models is larger (up to 47%). If there is no true interaction effect, the simpler model almost always (up to 95%) generalizes better to the population.

## Discussion

We conducted a simulation study to examine the ability of two competing linear regression models to explain and generalize a finite population data set. We looked at linear regression models that correctly capture the data-generating mechanism, and include an interaction effect, as well as models that were misspecified and did not capture the interaction effect.

Our simulation showed that the misspecification can bias the regression estimates – leading to canceling out an existing simple effect, but also to an effect in the opposite direction of the true effect. The comparison of explained proportion of variance in the sample to population favored the simple effects model in most conditions. However, the comparison of the explained proportion of variance solely in the population almost always favored the interaction effect models. Which model is favored depends on the configuration of the true $\beta$’s. If no interaction is present and sample sizes are small the simple effects model is favored. Furthermore, the simple effects model is favored when all simple effects and the interaction effect have the same sign *and* the sample size is small.

The configuration of the true $\beta$s leads to the shape of the interaction. The shape of the interaction effect is an often overlooked property of interaction effects. Our simulation showed that the shape of the interaction effect is not only relevant for power (Baranger et al., 2023; Giner-Sorolla, 2018), when estimating an interaction effect, but is in particular relevant when a misspecified model is applied.

When the goal is to explain the sample data and there is a true synergistic interaction, the simpler model can adjust for the introduced bias rather well, by overestimating both simple effects. When the interaction is antagonistic and not modeled, true simple effects can disappear or even reverse. In both cases, the misspecification can lead to spurious intercepts.

When one tries to generalize findings from the sample to the larger population there are two things to consider. The first is the comparison between models in terms of overfit. In this comparison, there is a tendency for the generalization of the simpler, misspecified model to be more coherent and the difference between sample and population to be smaller than for the correctly specified model. Put simply, the simpler misspecified model exhibits a lower degree of overfitting. The exception is when antagonistic interactions are present, in this case the simpler misspecified model actually exhibits a higher degree of overfitting.

The second is the comparison between models in terms of generalization to the full population. In this comparison, the model estimating the interaction effect (very) often outperforms the misspecified model, and can explain the unseen data of the population better. For both of these comparisons, the shape of the interaction is relevant, as the variance is typically larger for the antagonistic compared to the synergistic interaction. For all these comparisons, buffering interactions are also included. This interaction type always leads to results somewhere between those for the synergistic and antagonistic interaction types.

To illustrate the implications of the results, let us look at fictional researcher Andy, to showcase how model misspecification can impact conclusions. Researcher Andy is interested in the emotion dynamics of depression. Researcher Andy has the theory that individuals with high levels of both positive and negative affect have a higher risk for developing depression. Let us assume that in our fictional example all this is indeed true, and additionally there is an interaction between positive affect and negative affect.

There are two different scenarios regarding the interaction, either it is (a) synergistic (the larger the product between these two affect states, the more the scores on depression will be increased); or (b) antagonistic (the larger the product between these two affect states, the more the scores on depression will be decreased). Researcher Andy conducted the study and collected data from many participants (say, 1000). Researcher Andy knows that the more flexible a model is, the more likely it is that it overfits the data, and read articles that were critical about the inclusion of interaction effects in linear regression models. Thus, Researcher Andy decides to use a model that only includes the simple effects of positive and negative affect as predictors. Consider the following two scenarios.

*Scenario A*. The analysis suggests that both positive affect and negative affect increase the risk of depression. Researcher Andy is satisfied because this model seems to explain the sample data quite well. Researcher Andy replicates the study multiple times with similar results and comes to the conclusion that the experience of either emotion increases the risk for depression.

*Scenario B*. The analysis suggests that positive affect increases the risk of depression (as expected), but counter to the expectation, negative affect decreases the risk of depression (see Table 2 for a showcase of how simple effects can flip in the case of misspecification). Researcher Andy finds a rather small $R^2$, invests effort into identifying additional influential variables but cannot reliably replicate the results. Consequently, Researcher Andy recommends avoiding positive affect states to decrease depression risk.

Scenario B is arguably worse than Scenario A, not only because the model fits worse and does not generalize well to the population level but also because the interpretation of regression estimates is misleading. Researcher Andy is not helped by increasing the study sample sizes, nor by replicating the study. To solve the issue, Researcher Andy should compare models rather than a priori discarding the interaction model.

These two hypothetical scenarios ignore other important factors in theory building, such as how to establish causality. However, these scenarios highlight the importance of the shape of the interaction effect, when applying a misspecified model, for the conclusions we make. Considering that in psychology we can generally assume that we do not capture all possible large influences and interdependencies between variables (average $R^2$ of 0.4, see e.g., Smedslund et al. (2022)), even replicated and/or cross-validated models should not be considered sufficient to build theory, however they might be considered as having a high predictive value.

While in an ideal setting a strong theory drives conducted research, in frequent research settings, such as exploratory and early-stage research, it is not possible to have such a strong theory because strong theories will only emerge through an iterative process (Borsboom et al., 2021). In those cases, researchers should focus on model selection criteria and the robustness of the resulting model. If a researcher conducts exploratory research and suspects an interaction to be present they may use bootstrap procedures or, in a Bayesian context, sampling from the posterior to estimate the stability of the obtained regression coefficients and through this also the stability of the shape of an interaction. Our results show that it is in particular harmful to not model antagonistic interaction effects, thus if the results clearly point to an antagonistic interaction effect, one can suggest an interaction effect, which can be followed up on in consecutive research.

Additionally, we have seen that cross-validation can help to estimate the $R^2_{p}$ (see Appendix), and that even when a substantial difference between the $R^2$ between the simple and interaction effect model was found, it can be helpful to look at the cross-validated $R^2$ for both models. If one obtains negative cross-validated $R^2$ values, one can conclude that the model estimated for the sample probably does not actually work well in the population at all, and hence strongly overfitted the sample data. In general, it is advisable to (1) rule out alternative explanations for an interaction effect, such as measurement artefacts (floor and ceiling effects, see Rohrer and Arslan (2021)); to (2) make sure the measurement is sufficiently reliable (Blake & Gangestad, 2020; Aguinis et al., 2017; Murphy & Russell, 2017); and to (3) ascertain that non-linearity is not the reason for a significant interaction effect (Belzak & Bauer, 2019; Busemeyer & Jones, 1983; Ganzach, 1998; Lubinski & Humphreys, 1990).

We stress that for the current simulation we investigated scenarios where we varied the phenomenon in the data, however in a realistic setting more things are likely to influence the generalizability, such as measurement error (Blake & Gangestad, 2020; Aguinis et al., 2017), different functional shapes of data (Wagenmakers et al., 2012), and range restriction (Aguinis et al., 2017). Other limitations of our study are the coverage of the simulated parameter space, the arbitrary selection of mean values, and the complexity of our data-generating mechanism. Thus, we cannot generalize beyond our set of parameters. Furthermore, we only applied linear regression to analyze the data, different analyses might yield different results. Acknowledging that the parameter space is limited in our study, we believe it is highly likely that similar results would be found for different parameter combinations because many parameters covered quite extreme scenarios such as very high correlations between *X* and *Z* or very high $\lambda$.

We did not look at the root mean squared error (RMSE) that is oftentimes used to measure the out of sample prediction accuracy. The reason for this is that in order to make the RMSE comparable between different conditions, we need to standardize it as

17 $$\begin{aligned} RMSE^* = \frac{RMSE}{SD(Y)}, \end{aligned}$$

which results in

18 $$\begin{aligned} RMSE^*= \sqrt{1-R^2}. \end{aligned}$$

This shows that it would merely be a transformation of $R^2$, and hence using the RMSE as an additional measure would not reveal additional insights.

In sum, this simulation study underscores the importance of having a strong theory when applying statistical models to data. Without having a strong theory and without being able to capture all variables and inter-dependencies between variables this might result in a model that seems satisfactory when merely looking at explained variance or prediction errors, in sample as well as when generalized to the full population. However, even when these metrics seem to be good, interpreting regression weights, which is what we are ultimately interested in when building theory, poses the risk of making incorrect decisions that could have tremendous effects.

## Supplementary Information

Below is the link to the electronic supplementary material.

Supplementary file 1 (tex 11 KB)

## Data Availability

Materials and analysis code are available at https://osf.io/myq8u/.

## Appendix A Derivation noise level

$R^2$ is defined by

A1 $$\begin{aligned} R^2=\frac{Var(Y^*)}{Var(Y)} \end{aligned}$$

We have

A2 $$\begin{aligned} Var(Y)=&Var(Y^*+\varepsilon )=Var(Y^*)+Var(\varepsilon ) \nonumber \\ =&Var(Y^*)+\lambda ^2Var(Y^*) =(1+\lambda ^2)Var(Y^*), \end{aligned}$$

hence it follows that

A3 $$\begin{aligned} R^2=\frac{Var(Y^*)}{(1+\lambda ^2)Var(Y^*)}. =\frac{1}{1+\lambda ^2} \end{aligned}$$

From this expression, it is straightforward to derive

A4 $$\begin{aligned} \lambda =\sqrt{\frac{1}{R^2}-1}. \end{aligned}$$

## Appendix B Leave-one-out $R^2$ as a proxy for generalized $R^2$

In our simulation study, it was possible to generalize the regression models to the full population. However, this is not the case in applied settings. Thus, a proxy for the generalized $R^2$ is needed. There are different methods to assess the generalizability of models. We decided to use the cross-validation method leave-one-out (LOO; Tarpey (2000)) because of its ease to implement even with small sample sizes. The prediction error sum of squares (PRESS), is the sum of leave-one-out residuals $e_{(i)}$ squared, and can be used to compute the $R^2_{LOO}$ as follows:

B1 $$\begin{aligned} PRESS=\sum {e_{(i)}^2} \end{aligned}$$

B2 $$\begin{aligned} e_{(i)}=\frac{e_i}{1-h_{ii}} \end{aligned}$$

B3 $$\begin{aligned} R^2_{LOO}=1-\frac{PRESS}{\sum {(Y-\bar{Y})^2}} \end{aligned}$$

where $e_i$ are the residuals and $h_{ii}$ are the diagonal elements of $H=X(X'X)^{-1}X'$. *Y* indicates the observed values of the dependent variable, and $\bar{Y}$ is the mean of the dependent variable. First, we compare the $R^2_{LOO}$ to the $R^2(s)$ obtained on the sample. Second, we compare the $R^2_{LOO}$ to the generalized $R^2(p)$.

Table 5 shows the comparison of the $R^2_{LOO}$ obtained in the sample to the $R^2(p)$. It is visible that the regular $R^2_{LOO}$ tends to underestimate $R^2(p)$ with smaller sample sizes. However, with increasing sample sizes, this difference gets smaller. The differences between $R^2_{LOO}$ and $R^2(p)$ might seem large, however these differences are reasonably small when considering the differences between the regular $R^2(s)$ and $R^2(p)$.

**Table 5:** Comparison of the difference of $R^2_{LOO}$ and $R^2(p)$ for different models

	$R^2_{LOO}- R^2(p)_{simple}$			$R^2_{LOO}- R^2(p)_{interaction}$		
N	$Q_{0.10}$	Mdn	$Q_{0.90}$	$Q_{0.10}$	Mdn	$Q_{0.90}$
Synergistic				k = 2.048.000		
25	0.31	-0.07	0.21	-0.39	-0.06	0.25
50	0.16	-0.03	0.13	-0.19	-0.03	0.14
100	0.10	-0.02	0.09	-0.11	-0.01	0.09
250	-0.06	-0.01	0.05	-0.06	-0.01	0.05
500	-0.04	0.00	0.04	-0.04	0.00	0.04
1000	-0.03	0.00	0.03	0.03	0.00	0.03
Antagonistic				k = 2.048.000		
25	-0.36	-0.10	0.28	-0.42	-0.08	0.26
50	-0.19	-0.05	0.16	-0.21	-0.03	0.15
100	-0.12	-0.03	0.10	-0.12	-0.02	0.10
250	-0.07	-0.01	0.06	-0.07	-0.01	0.06
500	-0.04	-0.01	0.04	-0.05	0.00	0.04
1000	-0.03	0.00	0.03	-0.03	0.00	0.03
Buffering				k = 4.096.000		
25	-0.32	-0.07	0.22	-0.40	-0.07	0.25
50	-0.17	-0.04	0.14	-0.19	-0.03	0.14
100	-0.10	-0.02	0.09	-0.11	-0.02	0.09
250	-0.06	-0.01	0.06	-0.06	-0.01	0.06
500	-0.04	0.00	0.04	-0.04	0.00	0.04
1000	-0.03	0.00	0.03	-0.03	0.00	0.03
No Interaction				k = 1.024.000		
25	-0.29	-0.06	0.19	-0.38	-0.06	0.25
50	-0.16	-0.03	0.12	-0.18	-0.03	0.13
100	-0.10	-0.01	0.08	-0.10	-0.01	0.08
250	-0.06	-0.01	0.05	-0.06	-0.01	0.05
500	-0.04	0.00	0.04	-0.04	0.00	0.04
1000	-0.03	0.00	0.03	-0.03	0.00	0.03

## Declarations

### Open Practice Statement

Materials and analysis code are available at https://osf.io/myq8u/. The reported study was not preregistered.
